# Lipid Alterations in Early-Stage High-Grade Serous Ovarian Cancer

**DOI:** 10.3389/fmolb.2022.770983

**Published:** 2022-04-14

**Authors:** M. V. Iurova, V. V. Chagovets, S. V. Pavlovich, N. L. Starodubtseva, G. N. Khabas, K. S. Chingin, A. O. Tokareva, G. T. Sukhikh, V. E. Frankevich

**Affiliations:** ^1^ Federal State Budget Institution, National Medical Research Center for Obstetrics, Gynecology and Perinatology named after Academician V.I. Kulakov, Ministry of Healthcare of the Russian Federation, Moscow, Russia; ^2^ Federal State Autonomous Educational Institution of Higher Education, I.M. Sechenov First Moscow State Medical University of the Ministry of Health of the Russian Federation (Sechenov University), Moscow, Russia; ^3^ Department of Molecular and Chemical Physics, The Moscow Institute of Physics and Technology (National Research University), Moscow, Russia; ^4^ Jiangxi Key Laboratory for Mass Spectrometry and Instrumentation, East China University of Technology, Nanchang, China

**Keywords:** lipidome, mass spectrometry, omics technologies, oncolipids, serous ovarian cancer

## Abstract

Epithelial ovarian cancer (OC) ranks first in the number of deaths among diseases of the female reproductive organs. Identification of OC at early stages is highly beneficial for the treatment but is highly challenging due to the asymptomatic or low-symptom disease development. In this study, lipid extracts of venous blood samples from 41 female volunteers, including 28 therapy-naive patients with histologically verified high-grade serous ovarian cancer at different stages (5 patients with I-II stages; 23 patients with III-IV stages) and 13 apparently healthy women of reproductive age, were profiled by high-performance liquid chromatography mass spectrometry (HPLC-MS). Based on MS signals of 128 differential lipid species with statistically significant level variation between the OC patients and control group, an OPLS-DA model was developed for the recognition of OC with 100% sensitivity and specificity *R* ^2^ = 0.87 and Q^2^ = 0.80. The second OPLS-DA model was developed for the differentiation between I-II OC stages and control group with *R* ^2^ = 0.97 and Q^2^ = 0.86 based on the signal levels of 108 differential lipid species. The third OPLS-DA model was developed for the differentiation between I-II OC stages and III-IV stages based on the signal levels of 99 differential lipid species. Various lipid classes (diglycerides, triglycerides, phosphatidylchlorines, ethanolamines, sphingomyelins, ceramides, phosphatidylcholines and phosphoinositols) in blood plasma samples display distinctly characteristic profiles in I-II OC, which indicates the possibility of their use as marker oncolipids in diagnostic molecular panels of early OC stages. Our results suggest that lipid profiling by HPLC-MS can improve identification of early-stage OC and thus increase the efficiency of treatment.

## Introduction

Malignant ovarian tumors are among the most common oncogynecological diseases. Ovarian cancer (OC), 70% of which belongs to high-grade serous histotype (Amoroso et al., 2017), ranks first in the number of deaths among diseases of the female reproductive organs. In the Russian Federation the incidence of OC over the past 10 years has increased from 56.9 to 74.6 people per 100,000 population. The mortality rate of patients within a year after the diagnosis is 20.9 people per 100,000 population. Active detection has increased from 8.3 to 18.4 per 100,000 (Kaprin et al., 2020; Frederick Rand Ueland, 2021). However, in 2018, only 35.5% patients underwent radical surgery, while 60.4% patients underwent surgery with adjuvant chemotherapy (Kaprin et al., 2020). 25% of all cases are accompanied by resistance to treatment with platinum drugs, with a high risk of recurrence and progression (Li et al., 2017). According to various data, from 3 to 14% of the patients with diagnosed malignant neoplasm of the ovary are the women of reproductive age. More than 80% of them have an advanced stage of the disease (Ashraf and Dasari, 2010; Tomao et al., 2018). While population screening is not economically feasible, the identification of early OC stages (stages I-II according to the international classification of the International Federation of Gynecologists and Obstetricians—FIGO) is associated with improved outcomes of medical treatment. Unfortunately, the identification of early OC stages is associated with significant difficulties due to the asymptomatic or low-symptomatic development. Thus, it is urgent to develop new, more accurate diagnostic methods, because the prognosis is much more favorable when the disease is detected at early stages (Bekelman et al., 2014; Warren et al., 2018). Unfortunately, at present there are no adequate methods for the high-precision preoperative diagnosis of OC at early stages. Hence is the need to develop noninvasive and objective diagnostic methods.

One of the most promising approaches for the diagnosis of OC is high-performance liquid chromatography with mass spectrometry (HPLC-MS) metabolomics analysis, which combines chromatographic, spectrometric, and spectroscopic methods for sample analysis combined with statistical and bioinformatics processing of experimental data. Based on the results of metabolomics analysis, multiparameter models are developed to test the diagnostic accuracy of the identified biomarkers (Ray et al., 2017). A number of lipids display tumor-initiating activity, which is why they are colloquially called “oncolipids” (Ray et al., 2017). It has been shown that the diversity of oncolipids in the tumor microenvironment reflects the ability of those oncolipids to initiate and maintain invasion processes (Ray et al., 2017; Xu, 2018).

MS approaches for the determination of changes in blood lipidome are featured by short times of analysis and relative ease of sample preparation, as well as high information content of the obtained results. Characteristic mass spectra, also called “fingerprints,” are used to create a molecular classification model, which allows assignment of serum or plasma samples to a group of normal or pathological conditions. Here we studied the possibility of early (stages I-II) recognition of high-grade epithelial serous OC by lipidome profiling of blood plasma samples combined with statistical analysis of MS data.

## Materials and Methods

### Study Population

Patients undergoing cytoreductive surgery in the National Medical Research Center for Obstetrics, Gynecology and Perinatology named after Academician V.I. Kulakov (NMRC for OGP, Moscow, Russia) between November 2019 and July 2020 formed the study cohort. Case group included 28 patients with histologically verified high-grade serous ovarian cancer, stages I-IV according to FIGO. Control group included correspond controls (13 women).

Criteria for the inclusion of patients in the case group: histologically verified OC at I-IV stages according to FIGO; the age of patients from 18 to 55 years; informed consent to participate in the study. The absence of pathology in the control group was confirmed by standard clinical examination: questionnaire survey, clinical blood test and ultrasound examination of the pelvic organs.

Criteria for the exclusion of patients from the case group: primary multiple tumor process, histotypes (not high-grade serous OC), presence of *BRCA* mutation, comorbidities (diabetes mellitus, acute or chronic inflammatory process or infectious disease, pregnancy at the time of blood sampling.

**Compliance with ethical standards:** All patients signed an informed consent to participate in the study, approved by the Ethics Committee of the NMRC for OGP (Protocol No. 10 of 05 December 2019). We confirm that the study was conducted in accordance with the ethical standards of the institutional research committee, the Federal Laws of the Russian Federation (No 152, 323, 1,130 etc.,) and with the 1964 Helsinki Declaration.

### Sample Collection

Clinical samples were collected and analyzed. Blood plasma samples were collected in a tube containing EDTA before the administration of antibacterial, analgesic and other drugs before surgery. The collected blood samples were centrifuged for 20 min at 300 g and 4°C. After centrifugation, the supernatant was collected and centrifuged for 10 min at 12,000 g and room temperature. The resulting blood plasma was poured into 0.5 ml tubes. All tubes were accordingly labeled. Labeled tubes were frozen at -80°C.

### Extraction and Profiling of Lipids

Lipid extracts were prepared according to the modified Folch method. During this experiment, 480 μL of a chloroform-methanol mixture (2/1, v/v) was added to 40 μL of a plasma sample. The mixture was exposed to ultrasound for 10 min, after which 150 μL of water was added to the mixture. The mixture was then centrifuged for 5 min at 13,000 g and ambient temperature. An organic layer containing lipids was collected, subjected to vacuum drying, and then redissolved in a mixture of 100 μL isopropanol and 100 μL acetonitrile for subsequent MS analysis.

The lipid extracts were randomised and analyzed in triplicate on the Dionex UltiMate 3000 liquid chromatograph (Thermo Scientific, Germering, Germany) coupled to the Maxis Impact qTOF analyzer with electrospray ionization source (Bruker Daltonics, Bremen, Germany). 3 µl of the sample was injected onto a Zorbax XDB-C18 column (250 × 0.5 mm, 5 μm; Agilent, United States). Lipid separation was performed at a flow rate of 35 μL/min using water-acetonitrile (40/60, v/v) with 0.1% of formic acid and 10 mmol/L of ammonium formate as solvent A and isopropanol/acetonitrile/water (90/8/2, v/v/v) with 0.1% of formic acid and 10 mmol/L of ammonium formate as solvent B by a linear gradient from 30 to 95% (v/v) of solvent B over 25 min. The column temperature was 50°C. Mass spectra were obtained in the positive ion mode in the m/z range of 400–1,000 with the following settings: capillary voltage 4.1 kV, spray gas pressure 0.7 bar, drying gas flow rate 6 L/min, drying gas temperature 200°C.

Tandem MS experiments were run with the following parameters: three most intense peaks were selected after a full scan in the full mass range and were fragmented by collisional dissociation with energy of 35 eV. The time for excluding an ion from the analysis was 1 min.

### Data Processing and Lipid Identification

The raw HPLC-MS files were converted using the msConvert program from the Proteowizard 3.0.9987 package (Chambers et al., 2012) into the open MzXml format, containing information on the full MS, and into the ms2 format, containing information on tandem MS. The MzMine program (Pluskal et al., 2010) was also used to isolate peaks, normalize to the total ion current, and create a table containing information on the mass of the ion, the area of its chromatographic peak, and the retention time. Lipid identification was performed using LipidMatch scripts (Koelmel et al., 2017). The lipid nomenclature corresponds to LipidMaps (Sud et al., 2007).

**Statistical analyses** The processing of mass spectrometric data was performed using the multivariate discriminant analysis using orthogonal projections onto latent structures (OPLS-DA) (Trygg and Wold, 2002). OPLS-DA analysis was implemented using the “ropls” library (Thevenot et al., 2015). OPLS-DA models were created to classify samples according to clinical groups under study. Identified lipids were considered as independent variables in the models. The patient’s belonging to one of the studied groups served as a dependent variable. The quality of the developed models was determined by ROC-analysis including calculation of the area under the ROC curve, and calculating the threshold, sensitivity and specificity. The quality of the OPLS-DA models was also assessed by their ability to describe the variance of the analyzed data (R2) and predict possible new data (Q2). The Q2 parameter was calculated by 7-fold cross-validation. The model can be used to assign the analyzed samples to a particular clinical subgroup at Q2 ≥ 0.4 (Westerhuis et al., 2008; Worley and Powers, 2013). OPLS-DA models were tested for overfit using permutation test with 100 iterations. In addition, OPLS-DA was used to identify lipids that are most relevant for classification. This is done by analyzing the variable influence on projection VIP. Potential lipid markers included lipids with VIP >1 (Wold et al., 2001). The statistical significance of the difference in lipid levels between the study groups was carried out using the Mann-Whitney test with Benjamini–Hochberg correction. The results of the analysis were visualized using volcano plots, which allow to show the statistical significance and magnitudes of differences in lipid levels between groups. Score plots were used to represent the quality of OPLS-DA models and their ability to group samples according to clinical parameters.

Quantitative variables following a normal distribution were described using mean (M) and standard deviation (SD), 95% confidence interval (95% CI) for the mean were estimated. Quantitative variables following non normal distribution were described using median (Me) and lower and upper quartiles (Q1; Q3). The threshold significance level of *p*-value was taken equal to 0.05. For statistical processing of the results, scripts written in the R language version 3.3.3 (R Core Team, 2018) and the RStudio 1.383 program (R team, 2016), were used.

## Results

Patients of the study cohort and control group are comparable in age and body mass index (BMI; Table 1, Supplementary Table S1).

**TABLE 1:** Clinical data of patients of the study cohort and control group.

Clinical Groups		n (%)	Age, years					P
			Me	Q₁–Q₃	p	Me	Q₁–Q₃	
control group		13 (100)	49	45–52	0,355	49	45–52	0.067
**stages I-II, n = 5**	IA	2 (40.0)	54	52–55		55	54–55	
	IC	2 (40.0)				52	52–52	
	IIA	1 (20.0)				42	40–46	
**stages III-IV, n = 23**	IIIB	3 (13)	52	44–55		54	51–55	
	IIIC	17 (79.3)				48	46–50	
	IVA	3 (13)				42	40–46	

**Clinical groups**		**n (%)**	**BMI, kg/m2**					**P**
			**M ± SD**	**95% ДИ**	**P**	**M ± SD**	**95% ДИ**	
**control group**		13 (100)	24 ± 2	22–25	0,098	23	23–25	0.223
**stages I-II, n = 5**	IA	2 (40.0)	24 ± 1	23–25		24–25	24	
	IC	2 (40.0)				24–24	24	
	IIA	1 (20.0)				23	23–23	
**stages III-IV, n = 23**	IIIB	3 (13)	26 ± 3	24–27		25	23–25	
	IIIC	17 (79.3)				26	25–28	
	IVA	3 (13)				24	23–26	

**FIGURE 1:**
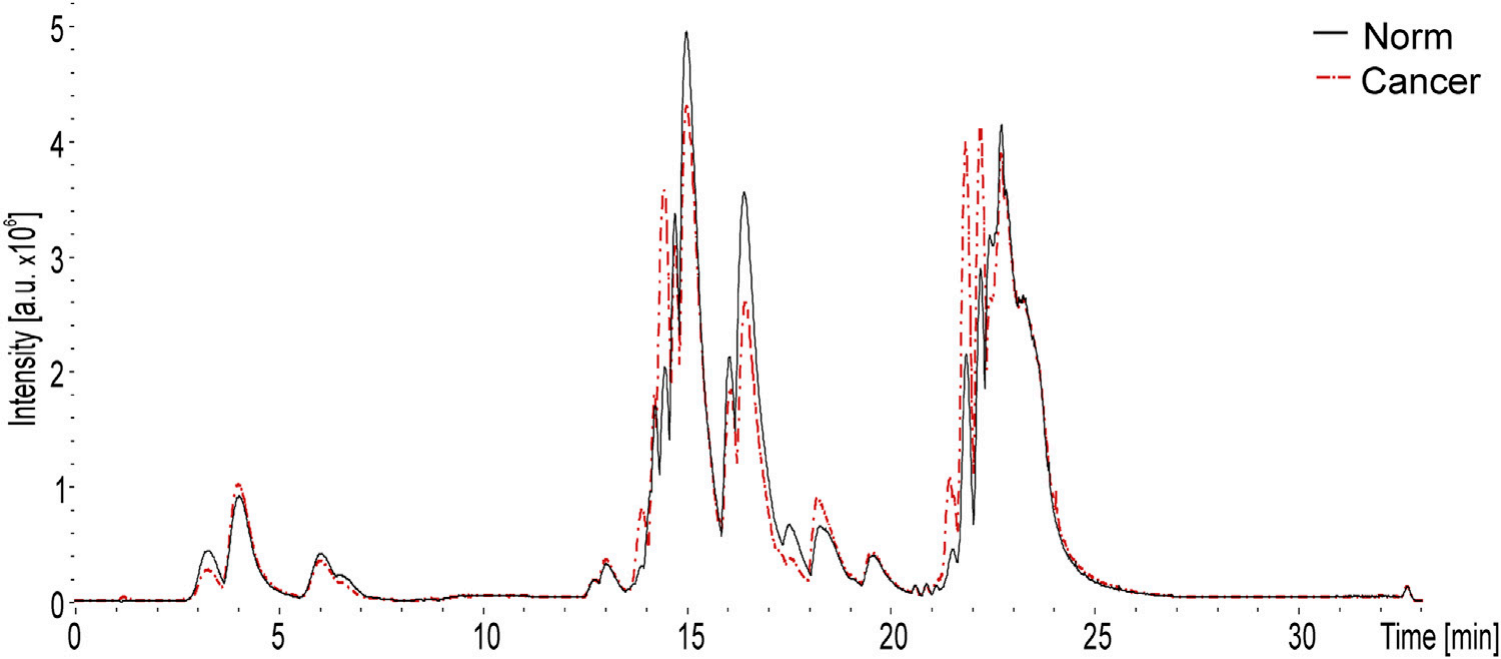
Typical total ion chromatogram of lipid ions recorded in the positive ion detection mode. The chromatogram was obtained by HPLC-MS analysis of the blood plasma extract. The red curve corresponds to the sample obtained from the patient with confirmed OC. The black curve corresponds to the sample from the patient from the control group.

A total of 345 lipid species were identified as a result of HPLC-MS analysis. Figure 1 shows a typical total ion current (TIC) chromatogram recorded in the positive ion detection mode from a lipid extract of blood plasma. The red curve corresponds to the samples obtained from patients with confirmed OC. The black curve corresponds to the samples from the control group. The signals with retention time between 0–10 min mainly belong to lysophospholipids, mono- and diglycerides. The signals with retention time between 10–20 min mainly belong to phospholipids. The signals with retention time >20 min mainly belong to triglycerides.

### Comparison of Patients of the Study Cohort and Control Group

**FIGURE 2:**
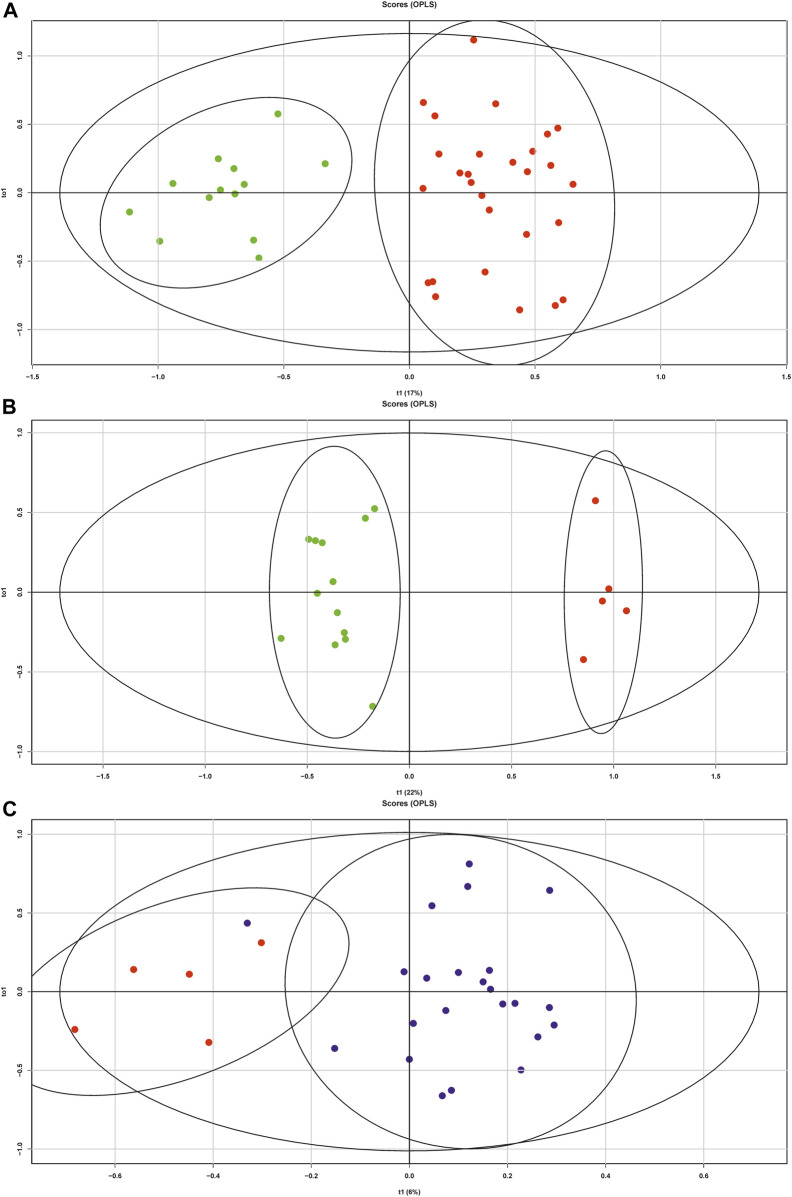
**(A)** Score plots based on the results of OPLS-DA analysis of MS data obtained in the positive ion mode for the blood plasma lipid extracts: **(A)** control group (green dots) and OC of stages I-IV (red dots); **(B)** control group (green dots) and OC of stages I-II (red dots); **(C)** OC of stages I-II (red dots) and stages III-IV (blue dots).

Figure 2A shows the score plot based on the OPLS-DA of HPLC-MS data obtained in the positive ion mode for the samples from the control group (green dots, n = 13) and samples from the patients with OC at I-IV stages (red dots, n = 28). The model explains the essential fraction of data using the latent variables, R2 was 0.87, and the Q2, which characterizes the part of data predicted by the model according to the cross validation, was 0.77 (Table 2). These values indicate the high quality of the model. The results of statistical validation of the OPLS-DA model by permutation analysis using 100 model permutations show that Q2 and R2 of the OPLS-DA model is higher than all the Q2 and R2 values in the permutation tests (Supplementary Figure S1A). These results demonstrated absence of the model overfitting, high goodness of fit and high predictive capability for the OPLS-DA model. The sensitivity and specificity of this OPLS-DA model reached 100% (Table 2). Out of the 345 identified lipid species, 109 lipid species with VIP >1 made a significant contribution to the constructed model (Supplementary Table S2). Mann-Whitney test revealed 147 lipid species with statistically significant difference between control group and OC of stages I-IV (Supplementary Table S3, Figure S2A).

**TABLE 2:** Parameters of the developed models.

Compa-red Groups	Model Type	Independent Variables	R2	Q2	AUC	Threshold	Sensitivity	Specificity
Control vs. OC stages I-IV	OPLS-DA	345 identified lipid species	0.87	0.77	1	0.57	1	1
Control vs. OC, stages I-II	OPLS-DA	345 identified lipid species	0.96	0.86	1	0.53	1	1
OC, stages I-II vs. III-IV	Logistic Regression	Cer-NS(d18:1/24:0); LPC(20:0); Plasmenyl-PE (P-18:0/20:3); PS(37:5)	-	-	1	0.5	1	1
		CL (28:0) (28:0); LPC(15:0); LPC(18:0); LPC(20:0)	-	-	1	0.5	1	1
		CL (28:0) (28:0); LPC(15:0); LPC(20:0); OxLPC(18:3(OOO))	-	-	1	0.5	1	1
		CL (28:0) (28:0); LPC(18:0); Plasmanyl-LPC(O-16:0); Plasmenyl-PC(P-16:1/18:0)	-	-	1	0.5	1	1

### Comparison of Patients of the Control Group and Patients With I-II Stages of OC

Figure 2B shows the score plot based on the OPLS-DA of HPLC-MS data obtained in the positive ion mode for the samples from the control group (green dots, n = 13) and samples from the patients with OC at I-II stages (red dots, n = 5). The developed model allows clear grouping of the data points into two clusters along the first main component. It is also characterized by high parameters, R2 = 0.96 and Q2 = 0.86, and passed permutation test (Supplementary Figure S1B). The sensitivity and specificity of the model also reached 100% (Table 2). There were 107 lipids with VIP >1 (Supplementary Table S4) and 100 lipids with *p*-value < 0.05 (Supplementary Table S5, Figure S2B).

### Comparison of Patients With I-II and III-IV Stages of OC

A score plot of the OPLS-DA model for OC group of stages I-II (red dots) and stages III-IV (blue dots) is shown in Figure 2C. Although the score plot shows effective clustering of data points and R2 = 0.72, the Q2 of the model is −0.22, and the permutation analysis (Supplementary Figure S1C) indicates a low quality of fit. To overcome this obstacle, logistic regression models were developed based on 21 lipid species with *p*-value < 0.05 (Supplementary Table S6). All combinations of independent variables were tested in the model creation and four of the models with the highest AUC were selected (Table 2). The final logistic regression models for differentiating stages I-II and III-IV included combinations of Cer-NS(d18:1/24:0), LPC(20:0), Plasmenyl-PE (P-18:0/20:3), PS(37:5) CL (28:0) (28:0), LPC(15:0), LPC(18:0), LPC(20:0), OxLPC(18:3(OOO)), Plasmanyl-LPC(O-16:0) and Plasmenyl-PC(P-16:1/18:0).

## Discussion

We performed untargeted metabolomics to make a step forward in better understanding the biological background of OC and to test the hypothesis that lipids were associated with OC- status. Using metabolomics technology, our aim was to identify biomarkers prospectively associated with early and advanced stages. The comparison of our data with earlier reports indicates several advantages of the proposed approach. M. Hilvo et al. found an increase in the level of hydroxybutyrates (2,4-dihydroxybutyrate, 3,4-dihydroxybutyrate) in the blood plasma of patients with high-grade serous OC compared with the blood samples of healthy women, and the prognostic value of this potential marker was also suggested (Hilvo et al., 2016; Braicu et al., 2017). However, the limitation of this and many other earlier studies is the predominance of patients with stages III-IV, which questions the application possibility of this method for diagnosis of early OC stages.

M. F. Buas et al. (2016) conducted a case-control study, over which they analyzed 50 blood plasma samples from patients who underwent surgical treatment for ovarian mass of serous histotype (OC or benign tumor—control group). Samples were examined using HPLC MS. It was found that the signal levels of 34 out of the 372 detected metabolites were statistically significantly different between the groups. The authors concluded that the revealed differences in the metabolic profile could improve the accuracy of the differential diagnosis of benign and malignant ovarian diseases (Buas et al., 2016). However, after exclusion patients with missing CA 125 values only 84 samples remained in the study (OC, n = 44; benign tumors in the control group, n = 40). Also, only postmenopausal women were included in the study, which should also be attributed to an important limitation of the study design. In addition, the use of the blood of patients with benign ovarian masses as control group does not exclude the “chimerism” of the lipid profile that can be changed due to other diseases. Therefore, more informative and accurate data could be obtained by comparing with the study group of patients, during the screening and diagnostic examination of which no data on ovarian masses were obtained (“healthy volunteers”). Similar to the study described above, in the study by Y. Hou et al. the control group consisted of patients with the presence of pathology of the reproductive organs: the comparison of blood lipid profiles was carried out between groups of patients with OC, benign ovarian lesions and uterine myoma (Hou et al., 2016). However, it was previously shown that each pathology used in the control group produces alterations in the lipid profile of blood. This fact should be considered when recruiting a control group. Nevertheless, the authors obtained important information about lipidome alterations: a decreased level of glycerolipids along with an increase of sphingolipids were clearly demonstrated in the blood of patients with OC (Hou et al., 2016).

In 2018 Niemi et al. studied lipidomic changes in early-stage (I-II) serous, mucinous and endometrioid ovarian tumours (Niemi et al., 2018) and concluded that different histologic type and clinical stage induce similar changes in lipid metabolism. The data obtained by Niemi et al. are, indeed, of great scientific and practical interest for research in this field. Observed in our study significant alterations (phosphatidylcholines (PC) and lysophosphatidylcholines (LPC)) appear to be similar to the ones reported in the mentioned research. The high clinical, pathological and molecular heterogeneity of ovarian tumors is largely responsible for the differences in the prognosis of treatment of patients with OC (Roberts et al., 2019),—so we would like to emphasize that we also selected patients for our cohort according to the principle of “one histotype”.

Another fundamental aspect that should be considered when planning a study devoted to the diagnosis of high-grade serous OC is the importance of two-step approach. At the first step a group of patients with a malignant process is revealed, which is radically different from the patients of the control group, consisting of examined women with excluded pathology. At the second step, an intragroup comparison (e. g., stages I-II vs stages III-IV) is necessary to distinguish between patients with early-stage OC and patients of the control group. This sequential approach allows differentiation of the early-stage high-grade serous OC from the advanced-stage ОС and the control group.

345 lipid species of eight groups were identified and analyzed: diglycerides, triglycerides, phosphatidylcholines, ethanolamines, sphingomyelins, ceramides, phosphatidylserines and phosphoinositols were positively associated with OC.

**FIGURE 3:**
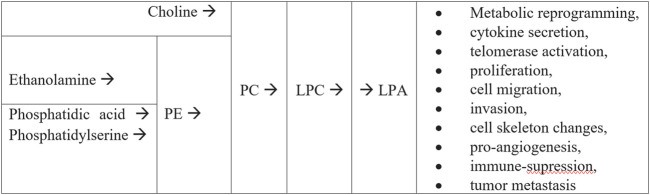
The Kennedy pathway (*de novo* synthesis of PE, PC and LPA from choline and phosphatidic acid) presenting the potential role of glycerophospholipids metabolism on cancerogenesis (according to Plewa et al. (Plewa et al., 2019)).

A vast majority of the significantly altered compounds identified in our research belongs to sphingolipids (ceramides, etc.,) and glycerophospholipids (glycerolipids, phosphatidylcholines, etc.,) (Supplementary Tables S2–6). In our study, we observed decreased plasma concentration of lipids in patients with early OC (Supplementary Figure S2B): Plasmanyl-LPC(O-16:0), Plasmenyl-PE (P-18:0/18:2), Plasmenyl-PE (P-18:0/20:3), Plasmenyl-PE (P-18:0/20:4), Plasmenyl-PE (P-18:1/22:6), Plasmenyl-PC(P-16:1/18:0), LPC(14:0), LPC(17:0), LPC(18:2), PS(37:5), SM(d20:0/18:4), Cer-NS(d18:1/24:0), including PC (PC(14:0_18:2), PC(18:2_22:6)). These features appear to be similar as was reported in the prospective study by Plewa et al. (2019). The global decrease of lipids can be partly explained by altered lipoprotein (high-density lipoprotein cholesterol and apolipoprotein A-1) levels (Braicu et al., 2017). The aberrant lipid metabolism of glycerophospholipids seems to be directly engaged in cell apoptosis or necrosis, cell proliferation as well as cellular signaling. The proposal of integration of the metabolic pathways potentially related to the role of glycerophospholipids metabolism in cancerogenesis involves Kennedy cycle (Figure 3). The PC and phosphatidylethanolamine (PE) are synthesized *de novo* from choline and ethanolamine, respectively. Phospholipids can serve as storage of energy in the form of fatty acyl chains, utilized only under specific conditions such as starvation and possibly also by energy-craving malignant cells (Bachmayr-Heyda et al., 2017). These multistep reactions constitute the two branches of the Kennedy Pathway. Furthermore, PC can be transformed into LPC by phospholipases A1 and A2. Then LPC can be converted into lysophosphatidic acid (LPA) by lysophospholipase D enzyme (lpD) (Xu et al., 2013). LPA in body fluids can consist of a mixture of various fatty acids (saturated and unsaturated), and thus it might play various roles in the body by activating different LPA receptors (Xu, 2018). As signal molecule LPC regulates inflammation, cell proliferation and the invasiveness of cancer cells. It serves as a substrate of lpD that converts LPC to LPA during cancer progression (Barber et al., 2012). LPA has been recently shown to be a pro-tumorigenic factor, involved in cell proliferation, differentiation, adhesion migration and invasion as well as promoting tumor cell survival and proliferation (Rogers et al., 2018; Plewa et al., 2019; Zhang et al., 2020). This phenomenon is accompanied by a deregulation of the homeostasis of the adhesion metabolism in stepwise progression of OC.

### Significance of the Study

HPLC-MS of blood lipid extract can allow detection of early-stage serous OC, which is very important for the OC treatment and prevention. We confirmed a dominant role of lipid alterations in OC. Considering ROC curve analysis results the following models: OPLS-DA (control vs. OC stages I-II, 345 identified lipid species) and logistic regression (OC stages I-II vs. III-IV) were characterized by a satisfactory AUC = 1.0. The integration of the proposed method into practical healthcare may improve the personalized algorithms for examination and the efficiency of patient treatment with malignant serous neoplasms of the ovaries.

### The Limitations of the Study

Our analyses point to a small set of features that were predictive of OC-case status, requiring further research and validation.

### Future Continuation of the Study

Revealed changes in the plasma lipidome of patients with OC appear to be products of disease progression and, therefore, could be useful diagnostic biomarkers for early detection of OC. It should be noted that due to the specificity of the lipid particle composition, it is possible that over 1,000 lipid species exist in a single eukaryotic cell. Moreover, they are geographically restricted within the cell (Van Meer et al., 2008). Therefore, to uncover the molecular background of the lipids involvement into cancerogenesis, further lipidomic research with special emphasis on cell, tissue and biofluid compartmentation of lipids are needed.

Taking into account such convergence with the cited studies, we believe that those lipid compounds are strongly correlated with OC-induced metabolic alterations and potentially involved in OC growth and progression. This finding could be a step forward to better understand the molecular background of OC initiation and progression.

## Conclusion

In present study, we have compared small-molecule features between OC cases (I-II vs. III-IV stages) and controls. Deep lipidome profiling of blood plasma by HPLC-MS combined with statistical analysis of MS data is demonstrated as a potentially powerful approach for the recognition of serous OC. This approach allows lipidome differentiation between plasma samples from early-stage and advanced-stage OC patients and control group. Our results indicate that HPLC-MS of blood lipid extracts can allow detection of early-stage serous OC, which is associated with significant difficulties using standard diagnostic methods. The integration of the proposed method into practical healthcare may improve the personalized algorithms for examination and treatment of ovarian high-grade serous carcinoma patients.

All materials used in current study were processed and stored in the biobank of the Federal State Budget Institution “National Medical Research Center for Obstetrics, Gynecology and Perinatology named after Academician V.I. Kulakov” Ministry of Health of Russia.

## Simple Summary

Epithelial ovarian cancer (OC) ranks first in the number of deaths among diseases of the female reproductive organs. Identification of OC at early stages is highly beneficial for the treatment but is highly challenging due to the asymptomatic or low-symptom disease development. In this study, EDTA-plasma samples are analyzed by untargeted high-performance liquid chromatography mass spectrometry, which provides more than 300 species. We demonstrate significant differences in the level of various lipid species, including diglycerides, triglycerides, phosphatidylcholines, ethanolamines, sphingomyelins, ceramides, phosphatidylserines and phosphoinositols, between the I-II stages OC, III-IV stages OC and healthy women with 100% sensitivity and specificity (*R* ^2^ = 0.87 and Q^2^ = 0.80). Our results suggest that lipid profiling of blood plasma by mass spectrometry can improve identification of early OC and thus increase the efficiency of treatment.

## Data Availability

The datasets presented in this study can be found in online repositories. The names of the repository/repositories and accession number(s) can be found below: MetaboLights, (MTBLS4454) (www.ebi.ac.uk/metabolights/MTBLS4454).
