# Supplementary material for: Lipid Alterations in Early-Stage High-Grade Serous Ovarian Cancer
Source: Front Mol Biosci. 2022 Apr 14;9:770983. doi: 10.3389/fmolb.2022.770983 (PMC9048792; doi:10.3389/fmolb.2022.770983)
Supplement: Supplementary file 1 [file Table1.DOCX]

**Lipid alterations in early-stage high-grade serous ovarian cancer**

M.V. Iurova^1,2^, V.V Chagovets^1^, S.V. Pavlovich^1,2^, N. L. Starodubtseva^1,3^, G.N. Khabas^1^, K.S. Chingin^4^, A.O Tokareva^1^, G.T. Sukhikh^1,2^, V.E. Frankevich^1^

^1^ Federal State Budget Institution “National Medical Research Center for Obstetrics, Gynecology and Perinatology named after Academician V.I. Kulakov” Ministry of Health of Russia.

^2^ Federal State Autonomous Educational Institution of Higher Education I.M. Sechenov First Moscow State Medical University of the Ministry of Health of the Russian Federation (Sechenov University)

^3^ The Moscow Institute of Physics and Technology (National Research University), Department of Molecular and Chemical Physics

^4^ Jiangxi Key Laboratory for Mass Spectrometry and Instrumentation, East China University of Technology, Nanchang 330013, P. R. China

**Keywords:** lipidome, mass spectrometry, omics technologies, oncolipids, serous ovarian cancer.

**

**

**Fig. S1.** Permutation test plots of OPLS-DA models based on lipids in plasma of patients in the groups under consideration: a) control group and stage I-IV OC; b) control group and stage I-II OC; c) stage I-II OC and stage III-IV OC

**
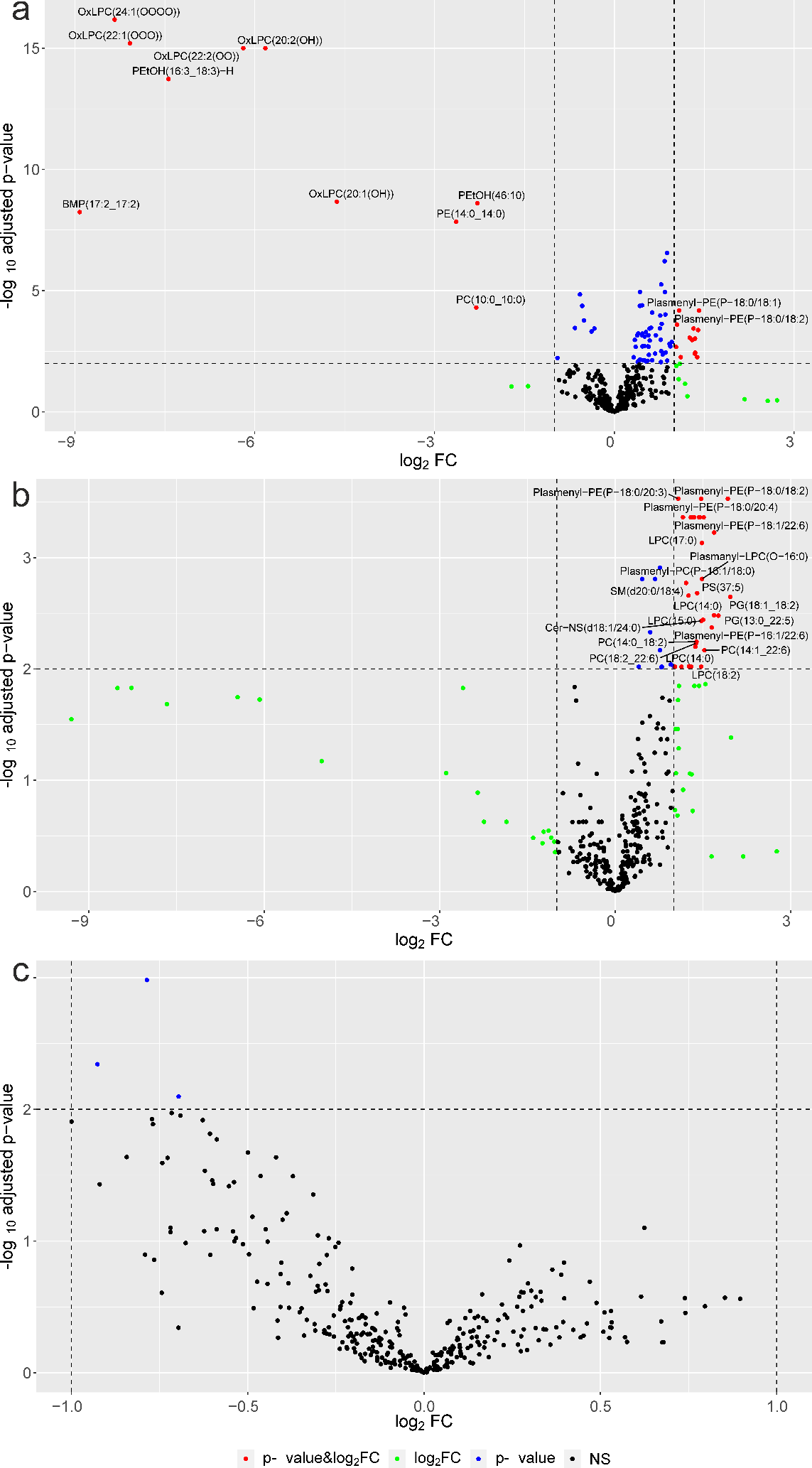
**

**Fig. S2.** Volcano plots based on a comparison of plasma lipid levels of patients in the groups under consideration: a) control group and stage I-IV OC; b) control group and stage I-II OC; c) stage I-II OC and stage III-IV OC

**Table S1.** Clinical data of patients of the study cohort and control group

| **Clinical groups** | **№** | **Stages FIGO** | **Age, years** | **BMI, kg/m^2^** |
| --- | --- | --- | --- | --- |
| **OC, stages I-II** | 1 | IA | 55 | 24,01 |
|  | 2 | IA | 52 | 23,14 |
|  | 3 | IC | 26 | 23,67 |
|  | 4 | IC | 54 | 24,54 |
|  | 5 | IIA | 55 | 24,8 |
| **OC, stages III-IV** | 6 | IIIB | 38 | 24,54 |
|  | 7 | IIIB | 42 | 21,55 |
|  | 8 | IIIB | 49 | 24,91 |
|  | 9 | IIIC | 37 | 26,93 |
|  | 10 | IIIC | 39 | 24,68 |
|  | 11 | IIIC | 55 | 26,81 |
|  | 12 | IIIC | 55 | 27,25 |
|  | 13 | IIIC | 51 | 25,46 |
|  | 14 | IIIC | 53 | 22,59 |
|  | 15 | IIIC | 55 | 25,86 |
|  | 16 | IIIC | 44 | 21,14 |
|  | 17 | IIIC | 55 | 29,72 |
|  | 18 | IIIC | 55 | 22,41 |
|  | 19 | IIIC | 51 | 24,68 |
|  | 20 | IIIC | 55 | 28,26 |
|  | 21 | IIIC | 55 | 28,88 |
|  | 22 | IIIC | 45 | 24,98 |
|  | 23 | IIIC | 55 | 25,65 |
|  | 24 | IIIC | 54 | 28,63 |
|  | 25 | IIIC | 53 | 29,41 |
|  | 26 | IVA | 48 | 28,26 |
|  | 27 | IVA | 52 | 24,01 |
|  | 28 | IVA | 44 | 21,56 |
| **Control group** | 29 | **-** | 45 | 23,14 |
|  | 30 |  | 42 | 26,81 |
|  | 31 |  | 49 | 21,14 |
|  | 32 |  | 54 | 21,55 |
|  | 33 |  | 53 | 28,88 |
|  | 34 |  | 38 | 26,93 |
|  | 35 |  | 52 | 23,14 |
|  | 36 |  | 48 | 22,59 |
|  | 37 |  | 51 | 22,59 |
|  | 38 |  | 45 | 23,32 |
|  | 39 |  | 48 | 24,3 |
|  | 40 |  | 52 | 20,58 |
|  | 41 |  | 54 | 24,68 |

**Table S2.** Lipids with VIP > 1, determined by OPLS-DA of lipidome data of patients with stage I-IV OC and control group

| **VIP** | **Lipid Name** |
| --- | --- |
| 2.69 | OxLPC(24:1(OOOO)) |
| 2.68 | OxLPC(22:1(OOO)) |
| 2.67 | PEtOH(16:3_18:3)-H |
| 2.64 | OxLPC(22:2(OO)) |
| 2.63 | OxLPC(20:2(OH)) |
| 2.44 | BMP(17:2_17:2) |
| 2.43 | Plasmenyl-PE(P-18:0/18:2) |
| 2.31 | PC(14:1_22:6) |
| 2.21 | PC(16:1_18:3) |
| 2.18 | PC(14:0_18:3) |
| 2.16 | PC(14:0_18:2) |
| 2.15 | PC(14:0_16:0) |
| 2.13 | Plasmenyl-PE(P-18:0/18:1) |
| 2.09 | PG(13:0_22:5) |
| 2.09 | PG(18:1_18:2) |
| 2.09 | PEtOH(46:10) |
| 2.02 | Plasmenyl-PE(P-18:0/20:4) |
| 1.90 | LPC(18:2) |
| 1.89 | LPC(18:0) |
| 1.84 | Plasmenyl-PC(P-16:1/18:0) |
| 1.84 | LPC(20:2) |
| 1.83 | Plasmanyl-LPC(O-16:0) |
| 1.81 | Plasmenyl-PE(P-18:1/22:6) |
| 1.81 | Plasmenyl-PE(P-18:1/20:4) |
| 1.78 | TG(10:0_16:0_18:2) |
| 1.75 | TG(16:0_18:1_8:0) |
| 1.74 | OxLPC(17:1(OO)) |
| 1.73 | Plasmenyl-PE(P-16:1/22:6) |
| 1.71 | LPC(18:3) |
| 1.70 | Plasmenyl-PE(P-18:0/20:4) |
| 1.70 | TG(12:0_18:2_18:2) |
| 1.69 | PC(18:2_22:6) |
| 1.67 | TG(18:2_22:6_22:6) |
| 1.66 | Plasmanyl-LPC(O-16:1) |
| 1.65 | SM(d20:0/18:4) |
| 1.64 | SM(d18:1/12:0) |
| 1.64 | Plasmenyl-PE(P-18:0/20:3) |
| 1.61 | TG(10:0_14:0_16:0) |
| 1.61 | PS(37:5) |
| 1.61 | LPC(15:0) |
| 1.61 | TG(14:0_16:1_18:2) |
| 1.54 | PS(15:1_20:4) |
| 1.52 | LPC(17:0) |
| 1.48 | TG(18:2_20:5_22:6) |
| 1.48 | PE(16:0_22:6) |
| 1.48 | PC(14:0_22:6) |
| 1.47 | LPC(18:1) |
| 1.46 | LPC(17:0) |
| 1.45 | LPC(18:1) |
| 1.44 | TG(16:0_18:2_22:6) |
| 1.43 | TG(18:1_18:2_22:6) |
| 1.42 | SM(d18:1/18:0) |
| 1.40 | TG(18:1_18:3_22:6) |
| 1.40 | DG(18:1_22:6) |
| 1.37 | SM(d18:1/20:3) |
| 1.36 | Plasmenyl-PC(P-16:0/18:2) |
| 1.34 | SM(d18:2/14:0) |
| 1.34 | TG(16:1_18:0_18:1) |
| 1.34 | OxTG(18:2_18:2_18:3(OH)) |
| 1.34 | Plasmanyl-PC(O-18:0/18:2) |
| 1.33 | TG(14:0_16:1_18:1) |
| 1.33 | TG(12:0_16:1_18:2) |
| 1.32 | PC(16:1_22:6) |
| 1.31 | MGDG(14:1_15:1) |
| 1.30 | CerP(d18:0/24:0) |
| 1.28 | OxTG(16:0_18:2_18:3(OH)) |
| 1.28 | TG(14:0_16:1_18:2) |
| 1.28 | OxLPC(18:4(OOOO)) |
| 1.28 | LPC(16:1) |
| 1.27 | TG(16:1_18:0_18:1) |
| 1.27 | TG(18:0_18:1_18:1) |
| 1.26 | CE(18:4) |
| 1.26 | PC(15:0_18:2) |
| 1.26 | PC(18:0_20:2) |
| 1.25 | PEtOH(20:0_22:5)-H |
| 1.25 | LPC(16:0) |
| 1.25 | PEtOH(20:1_22:2)-H |
| 1.25 | CE(18:3) |
| 1.23 | LPC(20:0) |
| 1.22 | PC(18:0_20:5) |
| 1.22 | SM(d22:4/20:0) |
| 1.21 | OxTG(18:1_18:3_18:3(OOO)) |
| 1.20 | TG(16:0_18:0_20:3) |
| 1.19 | Plasmanyl-PC(O-18:0/18:3) |
| 1.19 | PI(36:4) |
| 1.18 | LPC(16:0) |
| 1.16 | PC(16:1_22:6) |
| 1.16 | LPC(16:1) |
| 1.15 | CE(18:3) |
| 1.14 | PI(36:3) |
| 1.13 | Plasmenyl-PE(P-18:0/22:6) |
| 1.12 | TG(14:0_16:0_18:1) |
| 1.12 | PEtOH(20:0_20:1)-H |
| 1.12 | OxLPC(15:0(COOH)) |
| 1.12 | Plasmanyl-PC(O-22:0/20:3) |
| 1.11 | SM(d22:5/26:0) |
| 1.10 | Plasmanyl-LPC(O-18:1) |
| 1.08 | PE(18:4_22:5) |
| 1.07 | LPC(20:3) |
| 1.07 | Plasmenyl-PE(P-18:1/22:6) |
| 1.06 | OxTG(18:2_18:3_18:3(OOO)) |
| 1.04 | PC(18:0_18:1) |
| 1.03 | PEtOH(20:0_20:1)-H |
| 1.03 | PC(18:1_18:2) |
| 1.03 | OxTG(16:1_18:1_18:3(OOO)) |
| 1.02 | TG(16:0_18:1_22:6) |
| 1.01 | CE(20:2) |
| 1.01 | OxLPC(18:4(OOOO)) |

**Table S3.** Lipids with p-value < 0.05, determined by Mann-Whitney test of lipidome analysis data of patients with stage I-IV OC and control group

| **Lipid Name** | **Control Group** | **OC, stages I-IV** | **p-value** | **p adjusted** |
| --- | --- | --- | --- | --- |
| BMP(17:2_17:2) | 3e-05 (2e-05; 3e-05) | 0.01329 (0.00824; 0.01904) | < 0.001 | < 0.001 |
| CE(18:0) | 0.01375 (0.01255; 0.01478) | 0.01083 (0.00921; 0.01306) | 0.001 | 0.006 |
| CE(18:2) | 0.01336 (0.01255; 0.01517) | 0.01161 (0.01026; 0.01296) | 0.016 | 0.045 |
| CE(18:2) | 0.01225 (0.00966; 0.01334) | 0.00909 (0.00611; 0.01267) | 0.049 | 0.107 |
| CE(18:3) | 0.01372 (0.01047; 0.01733) | 0.00644 (0.00206; 0.01031) | 0.014 | 0.04 |
| CE(18:4) | 0.01091 (0.00867; 0.01372) | 0.00577 (0.00449; 0.01194) | 0.027 | 0.067 |
| CE(20:2) | 0.0138 (0.01154; 0.01648) | 0.01034 (0.00739; 0.01308) | 0.002 | 0.008 |
| Cer-NS(d18:1/24:0) | 0.01203 (0.0106; 0.01402) | 0.00752 (0.00398; 0.01254) | 0.012 | 0.036 |
| Cer-NS(d18:2/26:2) | 0.01338 (0.01056; 0.01422) | 0.01004 (0.00608; 0.01284) | 0.013 | 0.039 |
| CerP(d18:0/24:0) | 0.01004 (0.00881; 0.01065) | 0.01339 (0.01128; 0.01508) | < 0.001 | < 0.001 |
| DG(18:1_22:6) | 0.00693 (0.00513; 0.00788) | 0.00942 (0.00715; 0.01257) | 0.008 | 0.027 |
| LPC(14:0) | 0.00243 (0.0016; 0.00301) | 0.00097 (0.00082; 0.00113) | < 0.001 | 0.001 |
| LPC(15:0) | 0.01489 (0.01123; 0.01944) | 0.00862 (0.00671; 0.01148) | 0.002 | 0.009 |
| LPC(16:0) | 0.01364 (0.01257; 0.0165) | 0.01073 (0.00876; 0.01255) | < 0.001 | 0.001 |
| LPC(16:1) | 0.01206 (0.01121; 0.01637) | 0.00975 (0.00834; 0.01165) | 0.004 | 0.015 |
| LPC(17:0) | 0.01681 (0.01383; 0.01898) | 0.00833 (0.00567; 0.01207) | 0.001 | 0.003 |
| LPC(18:0) | 0.01688 (0.01447; 0.0187) | 0.0097 (0.00733; 0.01137) | < 0.001 | < 0.001 |
| LPC(18:1) | 0.01478 (0.01286; 0.01689) | 0.01032 (0.00871; 0.01183) | < 0.001 | 0.001 |
| LPC(18:2) | 0.01745 (0.01322; 0.01832) | 0.00983 (0.00717; 0.0115) | < 0.001 | < 0.001 |
| LPC(18:3) | 0.0143 (0.01095; 0.01997) | 0.00684 (0.00573; 0.00902) | 0.004 | 0.015 |
| LPC(20:0) | 0.01511 (0.01301; 0.01722) | 0.01061 (0.00823; 0.01252) | 0.002 | 0.008 |
| LPC(20:2) | 0.01808 (0.01479; 0.01899) | 0.00987 (0.00753; 0.01095) | < 0.001 | < 0.001 |
| LPC(20:4) | 0.01186 (0.01122; 0.01417) | 0.01133 (0.00856; 0.01257) | 0.047 | 0.104 |
| LPC(22:6) | 0.00878 (0.00687; 0.01061) | 0.01222 (0.00954; 0.01376) | 0.018 | 0.049 |
| MGDG(14:1_15:1) | 0.01511 (0.01258; 0.01664) | 0.00921 (0.00687; 0.01208) | < 0.001 | < 0.001 |
| MGDG(16:1_21:0) | 0.01255 (0.01185; 0.01331) | 0.01083 (0.00857; 0.01312) | 0.022 | 0.055 |
| MGDG(17:1_22:2) | 0.01071 (0.01029; 0.01143) | 0.01244 (0.01157; 0.01516) | 0.006 | 0.021 |
| MGDG(17:2_22:2) | 0.01269 (0.01179; 0.01392) | 0.01144 (0.00892; 0.01271) | 0.024 | 0.061 |
| OxLPC(15:0(COOH)) | 0.01403 (0.0122; 0.01618) | 0.01049 (0.00811; 0.01295) | 0.001 | 0.007 |
| OxLPC(17:1(OO)) | 0.01679 (0.01281; 0.01856) | 0.00939 (0.00763; 0.01176) | < 0.001 | 0.001 |
| OxLPC(18:4(OOOO)) | 0.01463 (0.01272; 0.01634) | 0.01063 (0.00845; 0.01152) | 0.004 | 0.015 |
| OxLPC(20:1(OH)) | 0 (0; 1e-05) | 0.00013 (7e-05; 0.00016) | < 0.001 | < 0.001 |
| OxLPC(20:2(OH)) | 0.00023 (0.00021; 0.00026) | 0.01425 (0.01269; 0.01753) | < 0.001 | < 0.001 |
| OxLPC(22:1(OOO)) | 6e-05 (5e-05; 7e-05) | 0.01529 (0.01387; 0.01694) | < 0.001 | < 0.001 |
| OxLPC(22:2(OO)) | 0.00018 (0.00017; 2e-04) | 0.01418 (0.01257; 0.0173) | < 0.001 | < 0.001 |
| OxLPC(24:1(OOOO)) | 5e-05 (3e-05; 5e-05) | 0.01491 (0.01379; 0.01678) | < 0.001 | < 0.001 |
| OxTG(18:1_18:1 _9:1(COOH)) | 0.01367 (0.01227; 0.01747) | 0.01068 (0.00843; 0.01284) | 0.014 | 0.041 |
| OxTG(18:2_18:2 _18:3(OH)) | 0.00678 (0.005; 0.00868) | 0.01197 (0.00722; 0.0174) | 0.012 | 0.036 |
| PC(10:0_10:0) | 1e-05 (1e-05; 1e-05) | 4e-05 (3e-05; 5e-05) | < 0.001 | < 0.001 |
| PC(14:0_22:6) | 0.01321 (0.01234; 0.01607) | 0.00678 (0.00497; 0.00991) | < 0.001 | 0.001 |
| PC(14:0_18:3) | 0.01871 (0.01171; 0.02757) | 0.00902 (0.0081; 0.01019) | 0.002 | 0.01 |
| PC(14:0_16:0) | 0.01796 (0.01207; 0.02578) | 0.00859 (0.00771; 0.00989) | 0.001 | 0.006 |
| PC(14:0_18:2) | 0.01928 (0.01307; 0.02437) | 0.00839 (0.00513; 0.00958) | < 0.001 | 0.001 |
| PC(14:1_22:6) | 0.01886 (0.0161; 0.02382) | 0.00565 (0.00456; 0.01033) | < 0.001 | < 0.001 |
| PC(15:0_18:2) | 0.01575 (0.0125; 0.01836) | 0.01017 (0.0081; 0.01275) | 0.002 | 0.008 |
| PC(16:0_16:0) | 0.01091 (0.0101; 0.01266) | 0.01312 (0.01084; 0.01453) | 0.01 | 0.031 |
| PC(16:0_22:6) | 0.01024 (0.00935; 0.01183) | 0.01265 (0.0111; 0.01417) | 0.017 | 0.045 |
| PC(16:0_20:4) | 0.0092 (0.00878; 0.0103) | 0.01299 (0.01111; 0.01439) | < 0.001 | < 0.001 |
| PC(16:1_22:6) | 0.00572 (0.00485; 0.01337) | 0.0133 (0.0123; 0.01432) | 0.003 | 0.012 |
| PC(16:1_18:3) | 0.01852 (0.01563; 0.02321) | 0.00659 (0.00458; 0.01006) | < 0.001 | < 0.001 |
| PC(16:1_18:2) | 0.01133 (0.01075; 0.01542) | 0.00918 (0.00681; 0.01167) | 0.027 | 0.067 |
| PC(17:0_18:2) | 0.01516 (0.0126; 0.0161) | 0.01085 (0.0088; 0.01305) | 0.016 | 0.044 |
| PC(18:0_18:1) | 0.01406 (0.0129; 0.01585) | 0.00919 (0.00805; 0.01279) | < 0.001 | 0.001 |
| PC(18:0_20:5) | 0.0148 (0.0137; 0.0162) | 0.01118 (0.00994; 0.01242) | < 0.001 | < 0.001 |
| PC(18:0_20:3) | 0.01289 (0.00993; 0.01639) | 0.00903 (0.00777; 0.01231) | 0.01 | 0.031 |
| PC(18:0_22:5) | 0.0133 (0.0111; 0.01428) | 0.01078 (0.0092; 0.01219) | 0.04 | 0.091 |
| PC(18:0_22:6) | 0.01171 (0.01103; 0.01387) | 0.0098 (0.00896; 0.01273) | 0.042 | 0.093 |
| PC(18:0_20:2) | 0.01521 (0.01234; 0.01646) | 0.00917 (0.00818; 0.01095) | < 0.001 | < 0.001 |
| PC(18:0_20:0) | 0.01417 (0.01323; 0.01449) | 0.01008 (0.0078; 0.01228) | 0.002 | 0.008 |
| PC(18:0_18:2) | 0.01708 (0.01535; 0.018) | 0.01173 (0.01028; 0.01311) | 0.014 | 0.04 |
| PC(18:0_20:4) | 0.01294 (0.01242; 0.01419) | 0.0111 (0.00997; 0.01276) | 0.006 | 0.021 |
| PC(18:1_20:5) | 0.01243 (0.01199; 0.01317) | 0.0109 (0.01045; 0.01193) | 0.003 | 0.013 |
| PC(18:1_18:2) | 0.01337 (0.01201; 0.01572) | 0.01054 (0.00952; 0.01215) | < 0.001 | 0.002 |
| PC(18:1_20:0) | 0.01251 (0.01145; 0.01295) | 0.00965 (0.00683; 0.01265) | 0.004 | 0.015 |
| PC(18:1_22:6) | 0.01229 (0.01091; 0.01382) | 0.01117 (0.00932; 0.01272) | 0.035 | 0.083 |
| PC(18:2_22:6) | 0.01627 (0.01422; 0.01827) | 0.0085 (0.00652; 0.01037) | < 0.001 | < 0.001 |
| PC(20:4_22:6) | 0.01357 (0.01158; 0.01527) | 0.00728 (0.00617; 0.01227) | 0.002 | 0.008 |
| PE(14:0_14:0) | 6e-05 (6e-05; 7e-05) | 0.00037 (0.00026; 0.00056) | < 0.001 | < 0.001 |
| PE(16:0_22:6) | 0.00576 (0.00477; 0.00749) | 0.01271 (0.00923; 0.01684) | 0.001 | 0.006 |
| PE(18:4_22:5) | 0.00893 (0.00846; 0.0108) | 0.0113 (0.0092; 0.0157) | 0.013 | 0.039 |
| PEtOH(16:3_18:3)-H | 1e-04 (7e-05; 0.00011) | 0.01468 (0.01318; 0.01726) | < 0.001 | < 0.001 |
| PEtOH(20:0_22:5)-H | 0.00707 (0.00463; 0.01328) | 0.01316 (0.01229; 0.0142) | 0.005 | 0.018 |
| PEtOH(20:0_20:1)-H | 0.01299 (0.01259; 0.01587) | 0.01061 (0.00958; 0.01176) | < 0.001 | 0.001 |
| PEtOH(20:1_22:2)-H | 0.01547 (0.01331; 0.01665) | 0.01072 (0.00989; 0.01219) | < 0.001 | < 0.001 |
| PEtOH(20:0_20:1)-H | 0.01315 (0.01254; 0.01497) | 0.01075 (0.00971; 0.01216) | < 0.001 | 0.001 |
| PEtOH(21:2_21:2)-H | 0.01241 (0.01193; 0.01291) | 0.01128 (0.01081; 0.0127) | 0.043 | 0.095 |
| PG(13:0_22:5) | 0.0161 (0.01311; 0.02701) | 0.00714 (0.00434; 0.01042) | 0.001 | 0.004 |
| PG(18:1_18:2) | 0.01636 (0.01294; 0.02373) | 0.00636 (0.00402; 0.01054) | 0.001 | 0.004 |
| PG(18:3_22:6) | 0.01634 (0.01236; 0.01808) | 0.01133 (0.00954; 0.01359) | 0.012 | 0.036 |
| Plasmanyl-LPC(O-16:0) | 0.01639 (0.01419; 0.01789) | 0.00913 (0.00504; 0.0109) | < 0.001 | < 0.001 |
| Plasmanyl-LPC(O-16:1) | 0.01745 (0.01429; 0.01857) | 0.00905 (0.00624; 0.01306) | < 0.001 | 0.001 |
| Plasmanyl-PC(O-18:0/18:3) | 0.0152 (0.01268; 0.01588) | 0.01107 (0.00855; 0.01247) | < 0.001 | 0.002 |
| Plasmanyl-PC(O-18:0/18:2) | 0.01519 (0.013; 0.01826) | 0.01037 (0.00797; 0.01297) | 0.001 | 0.004 |
| Plasmanyl-LPC(O-18:1) | 0.01484 (0.01152; 0.01891) | 0.00894 (0.00655; 0.01384) | 0.015 | 0.042 |
| Plasmanyl-PC(O-16:0/20:4) | 0.01461 (0.01305; 0.01701) | 0.01116 (0.00952; 0.01378) | 0.002 | 0.009 |
| Plasmanyl-PC(O-18:1/20:5) | 0.01352 (0.01251; 0.01478) | 0.01165 (0.01012; 0.01357) | 0.041 | 0.093 |
| Plasmanyl-PC(O-20:1/20:2) | 0.01418 (0.01226; 0.01667) | 0.01071 (0.00741; 0.01502) | 0.034 | 0.081 |
| Plasmanyl-PC(O-22:0/20:3) | 0.01718 (0.01131; 0.01817) | 0.01042 (0.00605; 0.01327) | 0.005 | 0.019 |
| Plasmanyl-PC(O-18:0/22:4) | 0.01368 (0.01182; 0.01499) | 0.01134 (0.00812; 0.01413) | 0.014 | 0.041 |
| Plasmenyl-PE(P-18:0/18:2) | 0.01893 (0.01646; 0.02324) | 0.00676 (0.00451; 0.00874) | < 0.001 | < 0.001 |
| Plasmenyl-PE(P-18:1/22:6) | 0.01662 (0.01376; 0.02333) | 0.00756 (0.0047; 0.01143) | < 0.001 | 0.002 |
| Plasmenyl-PE(P-16:1/22:6) | 0.01669 (0.01348; 0.02276) | 0.00741 (0.00487; 0.01128) | 0.003 | 0.013 |
| Plasmenyl-PE(P-18:1/22:6) | 0.01519 (0.01391; 0.01735) | 0.00915 (0.00657; 0.01262) | < 0.001 | 0.002 |
| Plasmenyl-PE(P-18:0/20:4) | 0.01481 (0.0139; 0.02115) | 0.00854 (0.0055; 0.0113) | < 0.001 | 0.002 |
| Plasmenyl-PE(P-18:0/18:1) | 0.01577 (0.01535; 0.02083) | 0.00837 (0.00634; 0.01052) | < 0.001 | < 0.001 |
| Plasmenyl-PE(P-18:0/22:6) | 0.01511 (0.01206; 0.01588) | 0.00981 (0.00699; 0.01247) | < 0.001 | 0.002 |
| Plasmenyl-PC(P-18:0/22:6) | 0.01479 (0.01364; 0.01723) | 0.01273 (0.00701; 0.01488) | 0.036 | 0.085 |
| Plasmenyl-PE(P-18:0/20:4) | 0.01671 (0.01508; 0.02091) | 0.00773 (0.00582; 0.01119) | < 0.001 | < 0.001 |
| Plasmenyl-PE(P-18:0/20:3) | 0.01664 (0.01319; 0.01704) | 0.00936 (0.00764; 0.01134) | < 0.001 | < 0.001 |
| Plasmenyl-PC(P-16:0/18:1) | 0.01452 (0.01226; 0.01554) | 0.011 (0.00855; 0.01341) | 0.002 | 0.009 |
| Plasmenyl-PC(P-16:1/18:0) | 0.01601 (0.01378; 0.02007) | 0.01006 (0.00733; 0.01173) | 0.001 | 0.004 |
| Plasmenyl-PC(P-16:0/18:2) | 0.01522 (0.01334; 0.01899) | 0.01054 (0.00837; 0.01288) | < 0.001 | 0.001 |
| Plasmenyl-PE(P-18:1/20:4) | 0.01903 (0.01114; 0.02219) | 0.00135 (0.00032; 0.01451) | 0.001 | 0.006 |
| PS(15:1_20:4) | 0.01469 (0.01207; 0.02151) | 0.00918 (0.00656; 0.01014) | 0.006 | 0.021 |
| PS(17:0_20:5) | 0.01433 (0.01308; 0.01588) | 0.01115 (0.01015; 0.01373) | 0.017 | 0.046 |
| PS(17:2_18:2) | 0.01294 (0.01198; 0.01677) | 0.01055 (0.00819; 0.01394) | 0.003 | 0.011 |
| SM(d16:1/16:0) | 0.01314 (0.01119; 0.01585) | 0.01015 (0.00708; 0.01255) | 0.006 | 0.021 |
| SM(d16:1/18:3) | 0.01397 (0.01129; 0.01577) | 0.01045 (0.00772; 0.0125) | 0.003 | 0.014 |
| SM(d18:1/18:0) | 0.00887 (0.00839; 0.01022) | 0.01364 (0.01108; 0.01576) | < 0.001 | < 0.001 |
| SM(d18:1/12:0) | 0.01434 (0.01082; 0.0193) | 0.00781 (0.00507; 0.00993) | < 0.001 | 0.002 |
| SM(d18:1/20:3) | 0.00956 (0.00901; 0.01064) | 0.01342 (0.01111; 0.01502) | < 0.001 | < 0.001 |
| SM(d18:1/18:1) | 0.01116 (0.01047; 0.01171) | 0.0142 (0.01227; 0.01626) | < 0.001 | < 0.001 |
| SM(d18:1/21:0) | 0.01248 (0.01056; 0.01518) | 0.01017 (0.00755; 0.01278) | 0.01 | 0.033 |
| SM(d18:1/18:1) | 0.01146 (0.01034; 0.01259) | 0.0135 (0.0107; 0.01496) | 0.025 | 0.061 |
| SM(d18:2/24:1) | 0.01106 (0.01035; 0.01171) | 0.01249 (0.01062; 0.01437) | 0.021 | 0.055 |
| SM(d18:2/22:0) | 0.01256 (0.01239; 0.01351) | 0.01101 (0.0093; 0.01307) | 0.005 | 0.018 |
| SM(d18:2/14:0) | 0.0146 (0.01364; 0.01591) | 0.00972 (0.00652; 0.01155) | < 0.001 | < 0.001 |
| SM(d18:2/18:1) | 0.011 (0.00972; 0.01133) | 0.01309 (0.01016; 0.016) | 0.008 | 0.027 |
| SM(d20:0/18:4) | 0.01666 (0.01412; 0.02167) | 0.00731 (0.0051; 0.01169) | < 0.001 | 0.002 |
| SM(d20:2/22:2) | 0.01231 (0.01195; 0.01367) | 0.01139 (0.00849; 0.01266) | 0.012 | 0.036 |
| SM(d22:0/20:5) | 0.01319 (0.013; 0.01364) | 0.01122 (0.00971; 0.01344) | 0.033 | 0.078 |
| SM(d22:4/20:0) | 0.00899 (0.00808; 0.00944) | 0.01366 (0.01121; 0.01806) | < 0.001 | < 0.001 |
| SM(d22:5/26:0) | 0.0074 (0.00592; 0.01) | 0.01106 (0.00865; 0.01645) | 0.007 | 0.024 |
| SM(d25:0/18:3) | 0.01398 (0.01224; 0.01569) | 0.01094 (0.00872; 0.01377) | 0.014 | 0.04 |
| TG(12:0_18:2_18:2) | 0.01508 (0.00979; 0.02294) | 0.00699 (0.00541; 0.00871) | 0.017 | 0.045 |
| TG(14:0_16:1_18:2) | 0.01338 (0.01068; 0.02137) | 0.00498 (0.00376; 0.00803) | 0.028 | 0.069 |
| TG(14:0_16:1_18:2) | 0.01255 (0.0105; 0.01744) | 0.00783 (0.00584; 0.00952) | 0.012 | 0.036 |
| TG(16:0_18:1_18:2) | 0.01158 (0.0105; 0.0119) | 0.01338 (0.0114; 0.01506) | 0.016 | 0.044 |
| TG(16:0_18:1_22:6) | 0.0099 (0.00851; 0.01117) | 0.01214 (0.00826; 0.01668) | 0.047 | 0.104 |
| TG(16:0_18:2_22:6) | 0.00682 (0.00534; 0.00856) | 0.00956 (0.00748; 0.01519) | 0.007 | 0.023 |
| TG(16:0_16:1_18:3) | 0.01298 (0.01136; 0.01557) | 0.00681 (0.0055; 0.01292) | 0.024 | 0.061 |
| TG(16:1_18:0_18:1) | 0.00314 (0.00291; 0.01733) | 0.01659 (0.0034; 0.02239) | 0.019 | 0.049 |
| TG(16:1_18:0_18:1) | 0.00657 (0.00591; 0.00888) | 0.01199 (0.00644; 0.02219) | 0.006 | 0.02 |
| TG(17:1_18:3_18:3) | 0.01146 (0.01045; 0.01268) | 0.01308 (0.01169; 0.01457) | 0.029 | 0.071 |
| TG(17:1_18:1_18:2) | 0.01418 (0.01173; 0.01538) | 0.01043 (0.00903; 0.01281) | 0.024 | 0.061 |
| TG(17:1_18:2_18:3) | 0.01065 (0.00921; 0.01128) | 0.01308 (0.01013; 0.01586) | 0.011 | 0.036 |
| TG(18:1_18:2_22:6) | 0.00621 (0.00563; 0.0081) | 0.01073 (0.0076; 0.01497) | 0.009 | 0.028 |
| TG(18:1_18:3_22:6) | 0.00571 (0.00435; 0.00687) | 0.00799 (0.00587; 0.01368) | 0.021 | 0.055 |
| TG(18:2_20:5_22:6) | 0.00218 (0.00192; 0.00384) | 0.00438 (0.00183; 0.00867) | 0.038 | 0.088 |
| TG(18:2_22:6_22:6) | 0.00233 (0.00183; 0.00552) | 0.00443 (0.00222; 0.00925) | 0.039 | 0.089 |
| PEtOH(46:10) | 0.00234 (0.00116; 0.0041) | 0.01135 (0.00939; 0.01705) | < 0.001 | < 0.001 |
| PI(36:2) | 0.01374 (0.01314; 0.01563) | 0.01088 (0.00796; 0.01371) | 0.023 | 0.058 |
| PI(36:3) | 0.01455 (0.01319; 0.01693) | 0.00933 (0.00849; 0.01192) | < 0.001 | 0.002 |
| PI(36:4) | 0.01484 (0.01442; 0.01608) | 0.01088 (0.0097; 0.01273) | < 0.001 | < 0.001 |
| PI(36:5) | 0.01337 (0.01221; 0.01606) | 0.01086 (0.00977; 0.01183) | 0.004 | 0.015 |
| PI(38:8) | 0.00956 (0.00881; 0.01073) | 0.01272 (0.01039; 0.01491) | 0.008 | 0.027 |
| PS(37:5) | 0.01554 (0.01271; 0.02226) | 0.00731 (0.0063; 0.01098) | 0.002 | 0.008 |

**Table S4.** Lipids with VIP > 1, determine by OPLS-DA of lipidome data of patients with stage I-II OC and control group

| **VIP** | **Lipid Name** |
| --- | --- |
| 2.62 | PEtOH(16:3_18:3)-H |
| 2.60 | OxLPC(22:2(OO)) |
| 2.59 | OxLPC(22:1(OOO)) |
| 2.59 | OxLPC(24:1(OOOO)) |
| 2.59 | BMP(17:2_17:2) |
| 2.58 | OxLPC(20:2(OH)) |
| 2.33 | Plasmenyl-PE(P-18:0/18:2) |
| 2.25 | PEtOH(46:10) |
| 2.20 | PG(18:1_18:2) |
| 2.14 | PG(13:0_22:5) |
| 2.11 | Plasmenyl-PE(P-18:0/20:4) |
| 2.11 | Plasmenyl-PE(P-18:1/22:6) |
| 2.05 | Plasmanyl-LPC(O-16:0) |
| 2.04 | Plasmenyl-PE(P-18:0/18:1) |
| 2.02 | Plasmenyl-PE(P-18:1/20:4) |
| 2.00 | LPC(18:0) |
| 1.99 | Plasmenyl-PE(P-16:1/22:6) |
| 1.99 | Plasmenyl-PE(P-18:0/20:4) |
| 1.93 | LPC(18:0) |
| 1.92 | PC(14:1_22:6) |
| 1.90 | PC(18:2_22:6) |
| 1.90 | PS(37:5) |
| 1.87 | TG(18:2_22:6_22:6) |
| 1.87 | SM(d20:0/18:4) |
| 1.86 | PC(16:1_18:3) |
| 1.86 | LPC(18:2) |
| 1.84 | Plasmenyl-PE(P-18:0/20:3) |
| 1.84 | Plasmanyl-LPC(O-16:1) |
| 1.83 | PC(14:0_18:2) |
| 1.83 | LPC(20:2) |
| 1.80 | Plasmenyl-PC(P-16:1/18:0) |
| 1.77 | LPC(17:0) |
| 1.75 | PS(15:1_20:4) |
| 1.73 | LPC(18:3) |
| 1.72 | LPC(15:0) |
| 1.68 | LPC(20:0) |
| 1.63 | OxLPC(17:1(OO)) |
| 1.58 | LPC(16:0) |
| 1.56 | TG(18:2_20:5_22:6) |
| 1.54 | LPC(18:1) |
| 1.54 | OxLPC(18:4(OOOO)) |
| 1.53 | Cer-NS(d18:1/24:0) |
| 1.50 | PC(14:0_16:0) |
| 1.49 | LPC(16:0) |
| 1.48 | SM(d18:1/12:0) |
| 1.48 | Plasmenyl-PC(P-16:0/18:2) |
| 1.46 | OxLPC(15:0(COOH)) |
| 1.46 | Plasmenyl-PC(P-24:1/22:6) |
| 1.44 | PC(14:0_18:3) |
| 1.43 | TG(18:1_18:2_22:6) |
| 1.43 | SM(d18:2/14:0) |
| 1.43 | LPC(18:1) |
| 1.41 | PC(15:0_18:2) |
| 1.38 | TG(16:0_18:2_22:6) |
| 1.37 | OxTG(18:1_18:3_18:3(OOO)) |
| 1.36 | Plasmenyl-PE(P-18:0/22:6) |
| 1.36 | Plasmanyl-PC(O-18:0/18:3) |
| 1.36 | PC(14:0_22:6) |
| 1.35 | Plasmenyl-PE(P-18:1/22:6) |
| 1.34 | PC(20:4_22:6) |
| 1.33 | PC(16:1_22:6) |
| 1.33 | TG(18:1_18:3_22:6) |
| 1.33 | MGDG(14:1_15:1) |
| 1.32 | CE(18:3) |
| 1.30 | DG(18:1_22:6) |
| 1.30 | Cer-NS(d18:2/26:2) |
| 1.29 | Plasmanyl-PC(O-18:0/18:2) |
| 1.26 | LPC(16:1) |
| 1.25 | CE(18:3) |
| 1.24 | TG(16:0_18:1_22:6) |
| 1.24 | PE(16:0_22:6) |
| 1.22 | Plasmenyl-PC(P-16:0/18:1) |
| 1.21 | Plasmanyl-PC(O-22:0/20:3) |
| 1.20 | OxTG(18:2_18:3_18:3(OOO)) |
| 1.19 | OxTG(16:1_18:1_18:3(OOO)) |
| 1.19 | PEtOH(20:1_22:2)-H |
| 1.17 | LPC(20:4) |
| 1.16 | OxLPC(18:4(OOOO)) |
| 1.15 | PC(16:1_22:6) |
| 1.14 | TG(16:1_18:0_18:1) |
| 1.14 | TG(12:0_18:2_18:2) |
| 1.14 | TG(16:1_18:0_18:1) |
| 1.12 | SM(d18:1/20:3) |
| 1.12 | PC(18:0_20:5) |
| 1.11 | SM(d18:1/18:0) |
| 1.11 | PEtOH(20:0_22:5)-H |
| 1.11 | OxLPC(18:3(OOO)) |
| 1.11 | PC(17:0_18:2) |
| 1.11 | TG(16:1_18:0_22:4) |
| 1.10 | CE(20:2) |
| 1.08 | OxTG(16:0_18:1_18:1(OOO)) |
| 1.07 | SM(d18:2/22:0) |
| 1.07 | TG(14:0_16:1_18:2) |
| 1.06 | PI(36:4) |
| 1.05 | Plasmanyl-LPC(O-18:1) |
| 1.05 | TG(18:0_18:1_18:2) |
| 1.04 | PG(18:3_22:6) |
| 1.04 | PC(18:0_20:0) |
| 1.03 | LPC(16:1) |
| 1.03 | CE(18:4) |
| 1.02 | PEtOH(15:0_15:0)-H |
| 1.02 | SM(d22:0/20:5) |
| 1.02 | PE(18:4_22:5) |
| 1.01 | Plasmanyl-PC(O-16:0/20:4) |
| 1.01 | TG(18:2_18:2_20:5) |
| 1.01 | CE(20:5) |

**Table S5.** Lipids with p-value < 0.05, determined by Mann-Whitney test of lipidome analysis data of patients with stage I-II OC and control group

| **Lipid name** | **Control group** | **OC, stages I-II** | **p-value** | **p adjusted** |
| --- | --- | --- | --- | --- |
| BMP(17:2_17:2) | 3e-05 (2e-05; 3e-05) | 0.01887 (0.01358; 0.0202) | 0.005 | 0.028 |
| CE(18:2) | 0.01336 (0.01255; 0.01517) | 0.01106 (0.00988; 0.01222) | 0.019 | 0.083 |
| CE(18:3) | 0.01561 (0.00967; 0.01984) | 0.00493 (0.00268; 0.00728) | 0.022 | 0.089 |
| Cer-NS(d18:1/24:0) | 0.01203 (0.0106; 0.01402) | 0.00401 (0.00308; 0.00491) | < 0.001 | 0.004 |
| Cer-NS(d18:2/26:2) | 0.01338 (0.01056; 0.01422) | 0.00613 (0.0057; 0.00669) | 0.012 | 0.057 |
| CL(28:0)(28:0) | 6e-05 (6e-05; 0.00023) | 5e-05 (4e-05; 6e-05) | 0.021 | 0.087 |
| LPC(14:0) | 0.00263 (0.00155; 0.00304) | 0.00059 (0.00045; 0.00091) | < 0.001 | 0.003 |
| LPC(15:0) | 0.01489 (0.01123; 0.01944) | 0.00551 (0.00306; 0.00638) | < 0.001 | 0.004 |
| LPC(16:0) | 0.01364 (0.01257; 0.0165) | 0.00625 (0.00501; 0.00776) | 0.002 | 0.014 |
| LPC(17:0) | 0.01681 (0.01383; 0.01898) | 0.00563 (0.00495; 0.00573) | < 0.001 | < 0.001 |
| LPC(18:0) | 0.01739 (0.01461; 0.01877) | 0.00606 (0.00482; 0.00613) | < 0.001 | < 0.001 |
| LPC(18:1) | 0.01478 (0.01286; 0.01689) | 0.00728 (0.00615; 0.00795) | 0.021 | 0.087 |
| LPC(18:2) | 0.01745 (0.01322; 0.01832) | 0.00598 (0.00527; 0.0062) | 0.001 | 0.01 |
| LPC(18:3) | 0.0143 (0.01095; 0.01997) | 0.00373 (0.0027; 0.00657) | 0.002 | 0.014 |
| LPC(20:0) | 0.01511 (0.01301; 0.01722) | 0.00737 (0.00584; 0.00826) | < 0.001 | < 0.001 |
| LPC(20:2) | 0.01808 (0.01479; 0.01899) | 0.00604 (0.00509; 0.00773) | < 0.001 | < 0.001 |
| LPC(20:4) | 0.01186 (0.01122; 0.01417) | 0.00769 (0.00611; 0.00857) | 0.008 | 0.043 |
| MGDG(14:1_15:1) | 0.01511 (0.01258; 0.01664) | 0.0092 (0.0089; 0.00923) | 0.005 | 0.031 |
| OxLPC(15:0(COOH)) | 0.01403 (0.0122; 0.01618) | 0.0061 (0.00574; 0.00947) | 0.008 | 0.043 |
| OxLPC(17:1(OO)) | 0.01679 (0.01281; 0.01856) | 0.00709 (0.00676; 0.01109) | 0.001 | 0.009 |
| OxLPC(18:4(OOOO)) | 0.01463 (0.01272; 0.01634) | 0.00862 (0.00707; 0.0099) | 0.001 | 0.01 |
| OxLPC(18:3(OOO)) | 0.0124 (0.01059; 0.01389) | 0.00754 (0.00727; 0.00869) | 0.011 | 0.057 |
| OxLPC(20:1(OH)) | 0 (0; 1e-05) | 0.00016 (0.00011; 0.00019) | 0.014 | 0.067 |
| OxLPC(20:2(OH)) | 0.00023 (0.00021; 0.00026) | 0.01771 (0.01746; 0.02265) | 0.003 | 0.019 |
| OxLPC(22:1(OOO)) | 6e-05 (5e-05; 7e-05) | 0.01804 (0.01701; 0.01871) | 0.002 | 0.015 |
| OxLPC(22:2(OO)) | 0.00018 (0.00017; 2e-04) | 0.01769 (0.01717; 0.02243) | 0.003 | 0.018 |
| OxLPC(24:1(OOOO)) | 5e-05 (3e-05; 5e-05) | 0.0178 (0.01677; 0.01883) | 0.002 | 0.015 |
| OxTG(18:1_18:1 _9:1(COOH)) | 0.01367 (0.01227; 0.01747) | 0.00924 (0.0092; 0.0099) | 0.019 | 0.084 |
| PC(10:0_10:0) | 1e-05 (1e-05; 1e-05) | 4e-05 (4e-05; 5e-05) | 0.033 | 0.129 |
| PC(14:0_22:6) | 0.01321 (0.01234; 0.01607) | 0.00671 (0.00489; 0.00792) | 0.02 | 0.086 |
| PC(14:0_18:3) | 0.01871 (0.01171; 0.02757) | 0.00925 (0.0083; 0.00966) | 0.003 | 0.019 |
| PC(14:0_16:0) | 0.01796 (0.01207; 0.02578) | 0.00858 (0.00777; 0.00901) | 0.001 | 0.01 |
| PC(14:0_18:2) | 0.01928 (0.01307; 0.02437) | 0.00735 (0.00473; 0.0088) | < 0.001 | 0.006 |
| PC(14:1_22:6) | 0.01886 (0.0161; 0.02382) | 0.0054 (0.0052; 0.00767) | 0.001 | 0.007 |
| PC(15:0_18:2) | 0.01575 (0.0125; 0.01836) | 0.00942 (0.00897; 0.01042) | 0.003 | 0.018 |
| PC(16:0_20:4) | 0.0092 (0.00878; 0.0103) | 0.0116 (0.01148; 0.0131) | 0.021 | 0.088 |
| PC(16:1_22:6) | 0.00572 (0.00485; 0.01337) | 0.01363 (0.01301; 0.01502) | 0.002 | 0.015 |
| PC(16:1_18:3) | 0.01852 (0.01563; 0.02321) | 0.00554 (0.00475; 0.00725) | 0.002 | 0.014 |
| PC(16:1_18:2) | 0.01133 (0.01075; 0.01542) | 0.00703 (0.00568; 0.01073) | 0.047 | 0.164 |
| PC(17:0_18:2) | 0.01516 (0.0126; 0.0161) | 0.01021 (0.00943; 0.01085) | 0.004 | 0.026 |
| PC(18:0_20:5) | 0.0148 (0.0137; 0.0162) | 0.01106 (0.01057; 0.01129) | 0.001 | 0.01 |
| PC(18:0_20:0) | 0.01417 (0.01323; 0.01449) | 0.01003 (0.0076; 0.01198) | 0.015 | 0.071 |
| PC(18:2_22:6) | 0.01627 (0.01422; 0.01827) | 0.00657 (0.00446; 0.00671) | < 0.001 | 0.006 |
| PC(20:4_22:6) | 0.01357 (0.01158; 0.01527) | 0.00656 (0.00566; 0.00712) | 0.006 | 0.035 |
| PE(14:0_14:0) | 6e-05 (6e-05; 7e-05) | 4e-04 (0.00037; 0.00064) | 0.02 | 0.086 |
| PEtOH(16:0_22:6) | 0.01474 (0.00937; 0.01701) | 0.01068 (0.00891; 0.01182) | 0.038 | 0.14 |
| PEtOH(16:3_18:3)-H | 1e-04 (7e-05; 0.00011) | 0.01561 (0.01371; 0.01881) | 0.003 | 0.021 |
| PEtOH(20:0_22:5)-H | 0.00707 (0.00463; 0.01328) | 0.01373 (0.01261; 0.01497) | 0.003 | 0.019 |
| PEtOH(20:1_22:2)-H | 0.01547 (0.01331; 0.01665) | 0.01046 (0.01037; 0.0107) | < 0.001 | 0.002 |
| PEtOH(20:1_20:1)-H | 0.01359 (0.00077; 0.01416) | 0.01532 (0.0149; 0.01581) | 0.016 | 0.071 |
| PG(13:0_22:5) | 0.0161 (0.01311; 0.02701) | 0.00503 (0.00435; 0.00663) | < 0.001 | 0.003 |
| PG(18:1_18:2) | 0.01636 (0.01294; 0.02373) | 0.00429 (0.00401; 0.00566) | < 0.001 | 0.002 |
| PG(18:3_22:6) | 0.01634 (0.01236; 0.01808) | 0.01111 (0.00851; 0.0119) | 0.027 | 0.108 |
| Plasmanyl-LPC(O-16:0) | 0.01639 (0.01419; 0.01789) | 0.00474 (0.00471; 0.0049) | < 0.001 | 0.002 |
| Plasmanyl-LPC(O-16:1) | 0.01745 (0.01429; 0.01857) | 0.00479 (0.0044; 0.00608) | 0.002 | 0.014 |
| Plasmanyl-PC(O-18:0/18:3) | 0.0152 (0.01268; 0.01588) | 0.00873 (0.00802; 0.01061) | < 0.001 | 0.002 |
| Plasmanyl-PC(O-18:0/18:2) | 0.01519 (0.013; 0.01826) | 0.00759 (0.0075; 0.01239) | 0.006 | 0.034 |
| Plasmanyl-LPC(O-18:1) | 0.01484 (0.01152; 0.01891) | 0.00817 (0.00752; 0.01229) | 0.05 | 0.172 |
| Plasmanyl-PC(O-16:0/20:4) | 0.01461 (0.01305; 0.01701) | 0.00968 (0.00963; 0.01391) | 0.034 | 0.131 |
| Plasmanyl-PC(O-22:0/20:3) | 0.01718 (0.01131; 0.01817) | 0.00712 (0.00667; 0.01276) | 0.019 | 0.084 |
| Plasmenyl-PE(P-18:0/18:2) | 0.01893 (0.01646; 0.02324) | 0.0041 (0.00393; 0.00719) | < 0.001 | < 0.001 |
| Plasmenyl-PE(P-18:1/22:6) | 0.01662 (0.01376; 0.02333) | 0.00553 (0.004; 0.00623) | < 0.001 | 0.001 |
| Plasmenyl-PE(P-16:1/22:6) | 0.01669 (0.01348; 0.02276) | 0.00553 (0.00499; 0.00594) | < 0.001 | 0.004 |
| Plasmenyl-PE(P-18:1/22:6) | 0.01519 (0.01391; 0.01735) | 0.00958 (0.00695; 0.01018) | 0.001 | 0.007 |
| Plasmenyl-PE(P-18:0/20:4) | 0.01481 (0.0139; 0.02115) | 0.00584 (0.00447; 0.00726) | < 0.001 | < 0.001 |
| Plasmenyl-PE(P-18:0/18:1) | 0.01577 (0.01535; 0.02083) | 0.00694 (0.00618; 0.00857) | < 0.001 | < 0.001 |
| Plasmenyl-PE(P-18:0/22:6) | 0.01511 (0.01206; 0.01588) | 0.00891 (0.00741; 0.00976) | 0.001 | 0.01 |
| Plasmenyl-PC(P-24:1/20:4) | 0.01131 (0.01041; 0.01732) | 0.00995 (0.00813; 0.01151) | 0.036 | 0.133 |
| Plasmenyl-PE(P-18:0/20:4) | 0.01671 (0.01508; 0.02091) | 0.00647 (0.00578; 0.00654) | < 0.001 | < 0.001 |
| Plasmenyl-PE(P-18:0/20:3) | 0.01664 (0.01319; 0.01704) | 0.00773 (0.0077; 0.00788) | < 0.001 | < 0.001 |
| Plasmenyl-PC(P-16:0/18:1) | 0.01452 (0.01226; 0.01554) | 0.00901 (0.00835; 0.01029) | < 0.001 | 0.005 |
| Plasmenyl-PC(P-16:1/18:0) | 0.01601 (0.01378; 0.02007) | 0.00811 (0.00739; 0.00822) | < 0.001 | 0.002 |
| Plasmenyl-PC(P-16:0/18:2) | 0.01522 (0.01334; 0.01899) | 0.00929 (0.00813; 0.01086) | < 0.001 | 0.001 |
| Plasmenyl-PC(P-24:1/22:6) | 0.01222 (0.0032; 0.01597) | 0.01633 (0.01412; 0.02231) | 0.034 | 0.131 |
| Plasmenyl-PE(P-18:1/20:4) | 0.01903 (0.01114; 0.02219) | 0.00174 (0.00027; 0.00355) | 0.008 | 0.041 |
| PS(15:1_20:4) | 0.01469 (0.01207; 0.02151) | 0.00689 (0.00666; 0.00691) | 0.001 | 0.009 |
| PS(17:0_20:5) | 0.01433 (0.01308; 0.01588) | 0.01107 (0.01046; 0.01264) | 0.039 | 0.142 |
| PS(17:2_18:2) | 0.01294 (0.01198; 0.01677) | 0.01151 (0.01039; 0.01199) | 0.012 | 0.059 |
| SM(d18:1/18:0) | 0.00887 (0.00839; 0.01022) | 0.01357 (0.01222; 0.01471) | 0.037 | 0.136 |
| SM(d18:1/12:0) | 0.01434 (0.01082; 0.0193) | 0.00805 (0.00626; 0.00965) | 0.001 | 0.01 |
| SM(d18:2/22:0) | 0.01256 (0.01239; 0.01351) | 0.00965 (0.00906; 0.011) | 0.013 | 0.063 |
| SM(d18:2/14:0) | 0.0146 (0.01364; 0.01591) | 0.00905 (0.00735; 0.01014) | 0.006 | 0.034 |
| SM(d20:0/18:4) | 0.01666 (0.01412; 0.02167) | 0.00698 (0.00512; 0.00869) | < 0.001 | 0.002 |
| SM(d20:2/24:1) | 0.01205 (0.01183; 0.01268) | 0.01099 (0.0078; 0.01106) | 0.043 | 0.152 |
| SM(d22:0/20:5) | 0.01319 (0.013; 0.01364) | 0.0096 (0.00839; 0.01047) | 0.005 | 0.03 |
| TG(12:0_18:2_18:2) | 0.01508 (0.00979; 0.02294) | 0.00537 (0.00527; 0.00654) | 0.031 | 0.122 |
| TG(15:0_18:1_18:2) | 0.01442 (0.01194; 0.01583) | 0.00879 (0.00727; 0.0111) | 0.044 | 0.153 |
| TG(17:1_18:1_18:2) | 0.01418 (0.01173; 0.01538) | 0.00991 (0.00971; 0.01288) | 0.036 | 0.133 |
| PEtOH(46:10) | 0.00234 (0.00116; 0.0041) | 0.01685 (0.01344; 0.01967) | 0.002 | 0.015 |
| PI(36:4) | 0.01484 (0.01442; 0.01608) | 0.0108 (0.01012; 0.01152) | 0.008 | 0.043 |
| PS(37:5) | 0.01554 (0.01271; 0.02226) | 0.00669 (0.00615; 0.0073) | < 0.001 | 0.002 |

**Table S6.** Lipids with p-value < 0.05, determine by Mann-Whitney test of lipidome analysis data of patients with OC (stages I-II vs III-IV)

| **Lipid Name** | **OC, stages I-II** | **OC, stages III-IV** | **p-value** | **p adjusted** |
| --- | --- | --- | --- | --- |
| Cer-NS(d18:1/24:0) | 0.00401 (0.00308; 0.00491) | 0.0084 (0.00678; 0.01257) | 0.012 | 0.496 |
| CL(28:0)(28:0) | 5e-05 (4e-05; 6e-05) | 6e-05 (5e-05; 0.00011) | 0.037 | 0.55 |
| LPC(15:0) | 0.00551 (0.00306; 0.00638) | 0.01074 (0.00728; 0.012) | 0.023 | 0.537 |
| LPC(16:0) | 0.00625 (0.00501; 0.00776) | 0.01112 (0.00961; 0.01269) | 0.023 | 0.537 |
| LPC(17:0) | 0.00563 (0.00495; 0.00573) | 0.00908 (0.00605; 0.01299) | 0.012 | 0.496 |
| LPC(18:0) | 0.00591 (0.00515; 0.00704) | 0.01016 (0.00887; 0.0116) | 0.012 | 0.496 |
| LPC(20:0) | 0.00737 (0.00584; 0.00826) | 0.0111 (0.00851; 0.01285) | 0.001 | 0.36 |
| LPC(20:2) | 0.00604 (0.00509; 0.00773) | 0.01038 (0.00902; 0.01156) | 0.017 | 0.531 |
| LPC(20:4) | 0.00769 (0.00611; 0.00857) | 0.01152 (0.01063; 0.01276) | 0.035 | 0.55 |
| OxLPC(18:4(OOOO)) | 0.00661 (0.00593; 0.00872) | 0.01138 (0.00936; 0.01203) | 0.038 | 0.55 |
| OxLPC(18:3(OOO)) | 0.00754 (0.00727; 0.00869) | 0.01197 (0.0105; 0.01283) | 0.036 | 0.55 |
| Plasmanyl-LPC(O-16:0) | 0.00474 (0.00471; 0.0049) | 0.01009 (0.00694; 0.01288) | 0.026 | 0.55 |
| Plasmenyl-PE(P-18:0/18:2) | 0.0041 (0.00393; 0.00719) | 0.00803 (0.00514; 0.00904) | 0.037 | 0.55 |
| Plasmenyl-PE(P-18:1/22:6) | 0.00553 (0.004; 0.00623) | 0.00828 (0.00525; 0.01183) | 0.013 | 0.496 |
| Plasmenyl-PE(P-16:1/22:6) | 0.00553 (0.00499; 0.00594) | 0.0078 (0.00544; 0.01162) | 0.011 | 0.496 |
| Plasmenyl-PE(P-18:0/20:4) | 0.00584 (0.00447; 0.00726) | 0.00935 (0.00653; 0.01218) | 0.011 | 0.496 |
| Plasmenyl-PE(P-18:0/20:3) | 0.00773 (0.0077; 0.00788) | 0.00948 (0.00782; 0.01206) | 0.032 | 0.55 |
| Plasmenyl-PC(P-16:1/18:0) | 0.00811 (0.00739; 0.00822) | 0.01019 (0.00732; 0.0118) | 0.023 | 0.537 |
| PS(15:1_20:4) | 0.00689 (0.00666; 0.00691) | 0.00937 (0.00676; 0.01117) | 0.032 | 0.55 |
| SM(d22:0/20:5) | 0.0096 (0.00839; 0.01047) | 0.0124 (0.00985; 0.01404) | 0.044 | 0.613 |
| PS(37:5) | 0.00669 (0.00615; 0.0073) | 0.00805 (0.00663; 0.01194) | 0.015 | 0.528 |
